# Characterization of a novel oxidase from *Thelonectria discophora* SANK 18292 involved in nectrisine biosynthesis

**DOI:** 10.1186/s13568-016-0176-1

**Published:** 2016-01-20

**Authors:** Ryuki Miyauchi, Hidetaka Sakurai, Yoichiro Shiba

**Affiliations:** New Modality Research Laboratories, R&D Division, Daiichi Sankyo Co., Ltd., 1-2-58, Hiromachi, Shinagawa-ku, Tokyo, 140-8710 Japan; Discovery Science and Technology Department, Drug Discovery and Biomedical Technology Unit, Daiichi Sankyo RD Novare Co., Ltd., 1-16-13, Kitakasai, Edogawa-ku, Tokyo, 134-8630 Japan; CM&C Planning Department, Pharmaceutical Technology Division, Daiichi Sankyo Co., Ltd., 1-12-1, Shinomiya, Kanagawa Hiratsuka-shi, 254-0014 Japan

**Keywords:** *Thelonectria discophora*, Fungi, Nectrisine, 4-Amino-4-deoxyarabinitol, Iminosugar, Oxidase

## Abstract

A fungus, *Thelonectria discophora* SANK 18292 (JCM 30947), produces nectrisine that has a nitrogen-containing heterocyclic 5-membered ring acting as a glycosidase inhibitor. Our previous study showed the possibility that 4-amino-4-deoxyarabinitol was enzymatically converted to nectrisine but the enzyme was not known. In order to characterize the enzyme, which is designated as NecC, it was purified from the fungus using ammonium sulfate precipitation and anion exchange chromatography. Liquid chromatography-tandem mass spectrometry analysis of NecC tryptic digests revealed partial NecC protein sequences. Subsequently, the partial DNA fragments were amplified by polymerase chain reaction with degenerate oligonucleotide primers and cloned. Then, *necC* complete genomic DNA was cloned by screening a genomic library of the fungus. Recombinant NecC also had NecC enzymatic activity, thus providing verification for the *necC* gene. NecC presumably belonged to the family of glucose methanol choline oxidoreductases, forming oligomers ranging approximately from 8 mer to 16 mer based on the results of native PAGE, and was also found to have a melting temperature of 57 °C, an optimal reaction condition of pH 7 at 30 °C, an activity inhibited by Cu^2+^ or ethylenediaminetetraacetic acid, and 4-amino-4-deoxyarabinitol as its preferred substrate. It was also indicated that not nectrisine but 4-amino-4-deoxyarabinitol was mainly extracted from the mycelium, and then was converted to nectrisine by the enzyme NecC in vitro. We believe that these findings are helpful to establish a nectrisine manufacturing process at large scale with the fungus.

**Electronic supplementary material:**

The online version of this article (doi:10.1186/s13568-016-0176-1) contains supplementary material, which is available to authorized users.

## Background

**Fig. 1:**
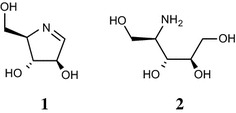
Structures of nectrisine (**1**) and 4-amino-4-deoxyarabinitol (**2**)

Nectrisine (**1**, also known as FR-900483, see Fig. 1) (Kayakiri et al. 1988) is an iminosugar that has a nitrogen-containing heterocyclic 5-membered ring showing inhibitory activity against α-glycosidase and other glycosidases (Shibata et al. 1988; Tsujii et al. 1996). Nectrisine can be biologically produced by *Nectria lucida* F-4490 (Shibata et al. 1988), which was treated as the synonym of *Thelonectria lucida* by Chaverri et al. (2011), and *Thelonectria discophora* SANK 18292 (JCM 30947) (Miyauchi et al. 2015), which was discovered in our screening program for nectrisine producing microorganisms. Species of *Thelonectria* are found on bark of recently dead or dying trees (Brayford et al. 2004; Chaverri et al. 2011). Recently, the biosynthetic pathway of nectrisine in *T. discophora* was studied by conducting incorporation experiments using ^13^C-labeled substrates (Miyauchi et al. 2015). In the study, a novel natural product, 4-amino-4-deoxyarabinitol (**2**) (Fig. 1), was isolated from the culture broth and it was indicated that 4-amino-4-deoxyarabinitol may be enzymatically converted to nectrisine. However, the genes and enzymes responsible for nectrisine biosynthesis have not yet been identified. Identification and characterization of the genes and enzymes are thus essential for developing an efficient manufacturing process of nectrisine using the fungus and furthermore, creating nectrisine highly producing microorganisms by genetic engineering.

The aim of the present study is to identify and characterize the enzyme and gene, namely NecC, from *T. discophora* that catalyzes the conversion of 4-amino-4-deoxyarabinitol to nectrisine. We report purification of the enzyme and identification of the DNA and cDNA of *necC*. Furthermore, we demonstrate that the enzyme forms oligomer and shows characteristics of the glucose-methanol-choline (GMC) oxidoreductase family. Finally, we suggest that not nectrisine but 4-amino-4-deoxyarabinitol could be mainly accumulated in *T. discophora* cells.

## Methods

### Fermentation of *T. discophora* and purification of 4-amino-4-deoxyarabinitol oxidase (NecC)

A nectrisine producer, *T. discophora* SANK 18292 (JCM 30947), (Miyauchi et al. 2015) which was deposited in the Japan Collection of Microorganisms, RIKEN BioResource Center (Tsukuba, Japan), was used in this study. The spore suspension was harvested from the cultures grown on potato dextrose agar (Wako Pure Chemical Industries, Osaka, Japan) slants, and were inoculated into A-1 medium (Miyauchi et al. 2015). The culture was incubated at 23 °C on a rotary shaker at 210 rpm for 5 days. Glucose/potato/yeast extract/calcium carbonate no. 3 (GPYC-3) medium containing 8 % glycerol, 1 % potato granule, 2.6 % yeast extract, 0.2 % CaCO_3_ was inoculated with the 1 % v/v culture and cultivated at 23 °C on a rotary shaker at 210 rpm for 4 days. The mycelium was harvested by centrifugation, and rinsed with a 0.9 % NaCl aqueous solution. The mycelium was frozen with liquid N_2_, then grinded with a pestle in a mortar, and resuspended with 50 mM sodium phosphate buffer, pH 7.0. The mycelium and debris were removed by centrifugation and filtration. The supernatant was subjected to ammonium sulfate fractionation at room temperature. The ammonium sulfate fraction precipitating between 0 and 30 % saturation was washed with 50 mM sodium phosphate, 5 mM dithiothreitol (DTT), 30 % saturation of ammonium sulfate buffer, pH 7.0 on ice. Then, the precipitate was dissolved in 50 mM sodium phosphate, 5 mM DTT buffer, pH 7.0 and dialyzed against the buffer with a Slide-A-Lyzer Dialysis Cassette (Life Technologies, Tokyo, Japan). The sample was applied to a 5 mL HiTrap DEAE FF anion exchange column (GE Healthcare, Tokyo, Japan) equilibrated with 50 mM sodium phosphate, 5 mM dithiothreitol buffer, pH 7.0 at a flow rate of 5 mL/min operated with an ACTAprime plus chromatography system (GE Healthcare) and linear gradient elution was performed in a range of 0–50 % of 50 mM sodium phosphate, 5 mM DTT, 1 M NaCl buffer, pH 7.0 for 15 min. Fractions showing 4-amino-4-deoxyarabinitol oxidase activity were recovered and adjusted to 50 % glycerol and stored at −20 °C.

Proteins were quantified with a DC Protein Assay Kit (Bio-Rad, Tokyo, Japan) according to the manufacturer’s instructions. Protein purity was confirmed by sodium dodecyl sulfate–polyacrylamide gel electrophoresis (SDS–PAGE) with molecular weight markers, Sea Blue Plus 2 Pre-Stained Standards (Life Technologies). The proteins were stained with Coomassie Brilliant Blue R-250 (CBB).

### Partial protein identification by liquid chromatography-tandem mass spectrometry (LC–MS/MS)

In-gel tryptic digestion and subsequent LC–MS/MS analysis with slight modifications were conducted to identify the protein as reported previously (Kubota et al. 2003; Ishizuka et al. 2010). The CBB-stained protein bands in SDS–PAGE were excised separately and were incubated in 50 mM NH_4_HCO_3_, pH 8.0 in 30 % CH_3_CN for 15 min at 37 °C to remove the CBB. Then, the gel pieces were dehydrated by soaking in CH_3_CN and subsequent evaporation. The proteins were reduced with 10 mM dithiothreitol, 20 mM NH_4_HCO_3_, pH 8.0 for 30 min at 50 °C and alkylated with 55 mM iodoacetamide, 20 mM NH_4_HCO_3_, pH 8.0 for 20 min at 20 °C in the dark. The gel pieces were evaporated thoroughly, and then incubated in digestion-buffer containing 50 μL of 20 mM NH_4_HCO_3_, 10 ng/μL trypsin (Sequencing Grade Modified Trypsin; Promega, Tokyo, Japan), and 0.005 % dodecyl-maltoside, pH 8.0 for 12 h at 37 °C. The resulting peptides were extracted once with 0.05 % formic acid in H_2_O and twice with 0.05 % formic acid in CH_3_CN. The collected extracts were evaporated to an amount of approximately 20 μL.

LC–MS/MS analyses were conducted using a LTQ-Orbitrap (Thermo Fisher Scientific, Yokohama, Japan) mass spectrometer and a DiNa nano-flow liquid chromatography system (KYA Technologies, Tokyo, Japan) equipped with a homemade BEH C_18_ (1.7 μm, Waters, Tokyo, Japan) electrospray ionization tip column (0.15 mm internal diameter × 50 mm length) and an Inertsil C_18_ (3 μm, GL Sciences, Tokyo, Japan) trap column (0.3 mm internal diameter × 1 mm length). The mobile phase consisted of solvent A, 0.1 % formic acid in H_2_O; and solvent B, 0.1 % formic acid in CH_3_CN. Elution of peptides was carried out at a flow rate of 150 nL/min with a linear gradient of 5–35 % of solvent B for 60 min at room temperature. The MS/MS spectra were searched in the National Center for Biotechnology Information non-redundant protein database using the Mascot program (Matrix Sciences, Tokyo, Japan). In addition, MS/MS spectra of major precursor ions unidentified by the Mascot search were manually inspected to assign amino acid sequences.

### Cloning of *necC* fragment

The fungal chromosomal DNA was isolated with DNeasy plant kits (Qiagen, Tokyo, Japan) from grinded frozen mycelium prepared as described above. Forward and reverse degenerate primers, AO1L-f: 5′-aaiisiccigaiggiaaigarwsigtitaygaygc-3′ and AO3L-r: 5′-acitaitaiatrtcrtgrtccatiarncc-3′, were designed from amino acid sequencing data of tryptic peptides to amplify an *necC* DNA fragment. PCR amplification was conducted using an Expand High Fidelity PCR System (Roche Diagnostics, Tokyo, Japan) and 16 μM of each of the primers with the following program: 94 °C for 2 min; 30 cycles of 94 °C for 15 s, 52 °C for 30 s, 72 °C for 75 s; and 72 °C for 7 min. The PCR product (717 bp) was gel-purified using Qiaquick Gel Extraction Kits (Qiagen) and its inner sequence was then amplified using the primers AO2L-f: 5′-taygtiggiggiccigtitaytgygtiggngg-3′ and AO3L-r with the following program: 94 °C for 2 min; 10 cycles of 94 °C for 15 s, 54 °C for 30 s, and 72 °C for 45 s; and 15 cycles of 94 °C for 15 s, 54 °C for 30 s, 72 °C for 45 s + 5 s per cycle; and 72 °C for 7 min. The gel-purified PCR product was cloned using the pDrive Cloning Vector in a Qiagen PCR Cloning Kit (Qiagen) and sequenced using a Dye Terminator Cycle Sequencing System with AmpliTaq DNA Polymerase (Life Technologies) and a 3730xl DNA Analyzer (Life Technologies).

### Construction and screening of the *T. discophora* genomic DNA library

The fungal chromosomal DNA was isolated from the grinded frozen mycelium with DNeasy plant kits (Qiagen) without using QIAshredder (Qiagen) and extracted with phenol–chloroform. The DNA (0.1 mg) was partially digested with two units of *Mbo*I at 37 °C for 4 min and dephosphorylated with calf intestinal alkaline phosphatase (CIAP). The genomic DNA fragments were ligated into *Xba*I-digested, dephosphorylated, and *Bam*HI-digested SuperCos1 cosmid vector (Agilent Technologies, Santa Clara, CA). Gigapack III Gold packaging extract (Agilent Technologies) was used for packaging the DNA according to the manufacturer’s instructions. *E. Coli* XL1-Blue MR (Agilent Technologies) was transfected by the resulting recombinant phage. Approximately 50,000 colonies from the genomic library blotted on Amersham Hybond-N+ membranes (GE Healthcare) were screened by hybridization with digoxigenin (DIG)-labeled partial *necC* DNA fragments using Dig Easy Hyb (Roche Diagnostics) at 43 °C for 14–16 h. The membranes were washed with 0.5 × SSC/0.1 % SDS at 55 °C. Detection was performed using CDP-Star (Roche Diagnostics). The positive cosmids were isolated, then digested with *Eco*RI and *Bam*HI separately and subcloned into pUC118 *Eco*RI/BAP vector or pUC118 *Bam*HI/BAP vector (Takara Bio, Shiga, Japan). The DNA fragments were sequenced as described above and assembled using Genetyx software (Genetyx, Tokyo, Japan).

### Bacterial expression of *necC* gene and purification of C-terminal His-tagged NecC

The total RNA of *T. discophora* was extracted and isolated using an RNeasy Plant Mini Kit (Qiagen) with an RNase-Free DNase Set (Qiagen) from the grinded frozen cells prepared as described above. The RNA was reverse transcribed using ReverTraAce-α- and Oligo (dT) 20 primer (Toyobo, Ohsaka, Japan) according to the supplier’s instructions. Then, *necC* cDNA was amplified by PCR using forward and reverse primers containing the restriction-enzyme site sequences *Nde*I-AO-f, 5′-agatatacacatatggatcatcttctccatatcgaca, and AO-*Sal*I-r, 5′-atagtcgacttgagcgtcgtttccaatcttc, and the polymerase Phusion High-Fidelity DNA Polymerase (New England Biolabs, Tokyo, Japan) with the following program; 98 °C for 30 s; 30 cycles of 98 °C for 10 s, 60 °C for 20 s, 72 °C for 60 s; and 72 °C for 7 min. The gel-purified PCR product, digested with *Nde*I and *Sal*I, was introduced into *Nde*I-*Sal*I-digested pET21b vector (Merck, Tokyo, Japan), generating the vector pET21bnecC. For expression of His-tagged NecC, the plasmid was transformed into *E coli* BL21 (DE3) (Merck). Cells were grown at 37 °C to an OD_600_ of 1.0 in shake flasks containing 2-YT medium (Life Technologies) with 100 mg/L ampicillin at 170 rpm, and then protein was expressed by induction with 0.4 mM isopropyl beta-d-thiogalactoside (IPTG), and the cultivation was then continued at 16 °C for 21 h at 170 rpm. Cells were pelleted by centrifugation, and resuspended in 100 mM sodium phosphate, 300 mM NaCl buffer, pH 7.8. The cells were freeze-thawed once, then disrupted with sonication on ice followed by centrifugation. For protein purification, the supernatant supplemented with 5 % glycerol was diluted tenfold with buffer A containing 20 mM sodium phosphate, 500 mM NaCl, 30 mM imidazole, pH 7.4 and applied to an Ni Sepharose column (HisTrap HP, GE Healthcare) at a flow rate of 5 mL/min operated with an AKTAexplorer 10S chromatography system (GE Healthcare). The absorbed protein was eluted with buffer B containing 20 mM sodium phosphate, 500 mM NaCl 500 mM imidazole, pH 7.4 using gradient elution, from 0 to 100 % B for 20 column volumes (CV), and 100 % B in 5 CV. The purified sample was dialyzed against phosphate buffered saline (PBS) (Takara bio).

### LC–MS analysis of nectrisine and 4-amino-4-deoxyarabinitol

To detect and efficiently separate nectrisine and 4-amino-4-deoxyarabinitol by HPLC, samples were labeled using 4-fluoro-7-nitrobenzofurazan (NBD-F) (Dojindo Laboratories, Kumamoto, Japan) (Watanabe and Imai 1981; Imai and Watanabe 1981). Cell extracts (20 μL) were reduced with 10 μL of NaBH_4_ solution (1 mg/mL) to convert nectrisine to 1,4-Dideoxy-1,4-imino-d-arabinitol (DAB). The resulting solutions were heated at 60 °C for 2 min with 60 μL of 2 g/L NBD-F in methanol and 10 μL of 100 mM borate buffer, pH 8.2. The solutions were cooled to room temperature and acidified with 10 μL of 0.5 M HCl aqueous solution. LC–MS analysis was conducted using an Acquity UPLC System and a Synapt G2-S Mass Spectrometer (Waters). NBD-labeled samples were injected into a Unison UK C18 4.6 × 150 mm column (Imtakt, Kyoto, Japan) at a flow rate of 1 mL/min at 30 °C. The mobile phase consisted of solvent A, 10 mM NH_4_CO_2_H/2.2 mM HCO_2_H in H_2_O; and solvent B, 2.2 mM HCO_2_H in CH_3_CN, using the gradient elution of, 10 % B for 8 min, from 10 to 90 % B for 20 min, and 90 % B for 2 min.

### Physicochemical analysis of NecC

NecC from *T. discophora* was used for physicochemical analysis. The absorption spectrum of NecC at a concentration of 1 mg/mL in PBS, pH 7.4 was recorded with a DU-730 Spectrophotometer (Beckman-Courter, Tokyo, Japan). To release the cofactor of the enzyme, 5 μL of trichloroacetic acid (TCA) (Wako Pure Chemical Industries) was added to 100 μL of the NecC solution, and incubated for 1 h at room temperature. Insoluble proteins were precipitated by centrifugation at 16,000*g* for 5 min. Then, the absorption spectrum of the supernatant was recorded. Flavin adenine dinucleotide (FAD) (Nacalai Tesque, Kyoto, Japan) dissolved into PBS containing 4.8 % TCA was used as a control molecule.

The oligomeric state of the native NecC was analyzed by blue native PAGE (Schägger and von Jagow 1991; Schägger et al. 1994) and size exclusion-high performance liquid chromatography (SE-HPLC). Blue native PAGE was performed on a NativePAGE 3–12 % gel (Life Technologies) with NativeMark Unstained Protein Standards (Life Technologies) as molecular weight markers and stained with CBB. SE-HPLC was performed on an LC2010 CHT HPLC system (Shimadzu, Kyoto, Japan) equipped with an Acquity UPLC BEH200 SEC column, 4.6 × 150 mm, 1.7 μm particle size (Waters). The mobile phase was 20 mM sodium phosphate, 0.2 M KCl buffer, pH 6.8. The flow rate was 0.2 mL/min. The column oven temperature was 40 °C. Absorbance at 280 nm was recorded.

Differential scanning calorimetry (DSC) was performed on a VP-Capillary DSC system (Malvern, Worcestershire, UK). The sample, diluted to 0.45 mg/mL with PBS, pH 7.4, was heated from 20 to 90 °C at a scanning rate of 60 °C/h. PBS, pH 7.4 was used as a reference buffer. A buffer scan was subtracted from the protein scan and normalized for the protein concentration.

### Enzyme assay

Activity of the native NecC was determined by measuring the rate of H_2_O_2_ production spectrophotometrically (Klei et al. 1990; Soldevila and Ghabrial 2001). The standard assay mixture consisted of 0.6 mM 2,2′-azino-bis (3-ethylbenzthiazoline 6-sulfonic acid) (ABTS) (Sigma-Aldrich, Tokyo, Japan), 3 units of peroxidase from horseradish, 1 mM of 4-amino-4-deoxyarabinitol, 47.1 mM of potassium phosphate, pH 7.0, and an appropriate amount of sample containing NecC in a final volume of 1 mL. After incubation at 30 °C for 10 min, the reaction was stopped by adding 0.1 mL of a 4 N HCl aqueous solution. Absorbance at 420 nm of the solution was measured with a spectrophotometer. One unit of enzyme activity was defined as the amount of enzyme catalyzing the oxidation of 2 μmol of ABTS per min under the conditions described above. Activities against d-sorbitol, d-arabinitol or xylitol were measured by replacing 4-amino-4-deoxyarabinitol with each sugar alcohol in the standard assay mixture.

### Accumulation of 4-amino-4-deoxyarabinitol in *T. discophora*

A *T. discophora* culture was filtered with filter aids, Radiolite #2000 (Showa Chemical Industry, Tokyo, Japan) then stored at 4 °C until use. Metabolites were extracted by heat treatment as follows. The cooled broth (100 g) was added to 400 mL of tap water previously maintained at 61 °C in an egg-plant shaped flask, and incubated at 51–54 °C for 70 min. Then, it was cooled immediately in an ice-water bath and filtered. The filtrate was stirred at 4 °C to convert 4-amino-4-deoxyarabinitol to nectrisine. To detect nectrisine and 4-amino-4-deoxyarabinitol directly, HPLC with evaporative light scattering detection (ELSD) was performed using an Agilent 1100 HPLC system (Agilent Technologies) equipped with an evaporative light scattering detector Model 300S (SofTA, Westminster, CO) and an Xbridge BEH HILIC column, 4.6 × 75 mm, 2.5 μm (Waters) (Kimura et al. 2004) under the following conditions: mobile phase, 90 % CH_3_CN/10 % H_2_O/20 mM CH_3_CO_2_NH_4_; flow rate, 1 mL/min; column oven temperature, 40 °C; spray chamber, 40 °C; and drift tube, optical cell, and exhaust tube, 60 °C.

### Nucleotide and amino acid sequence accession numbers

The nucleotide sequence was deposited in the EMBL/GenBank/DDBJ databases under the accession number LC056029.

## Results

### Purification of NecC

**Fig. 2:**
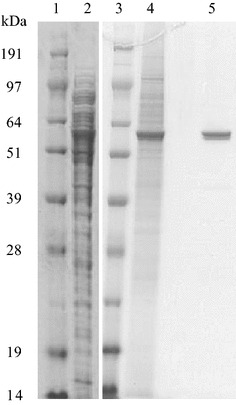
Purification of NecC from *T. discophora*. Reducing 12 % SDS–PAGE gels contain the following: *lanes 1* and *3* marker proteins; *lane 2* cell-free extract; *lane 4* 0–30 % ammonium sulfate fraction; *lane 5* purified NecC after DEAE-Sepharose column chromatography

NecC was purified from the extract of *T. discophora* based on the conversion activity of 4-amino-4-deoxyarabinitol to nectrisine. Almost 100 % of activity was recovered in a 0 % to 30 % ammonium sulfate fraction with 7.8-fold purification (Table 1). In the subsequent DEAE anion exchange chromatography, fractions that showed the activity were collected, resulting in a sixfold purification with a 25 % recovery yield from the extract. The purified NecC had a specific activity of 0.67 U/mg, and gave double bands for approximately 60 kDa in SDS–PAGE (Fig. 2).

**Table 1:** Purification of NecC from *T. discophora*

Purification step	Total protein (mg)	Total activity (U)	Specific activity (U/mg)	Yield (%)	Purification (fold)
Mycelium extract	363.9	40.7	0.11	100	1.0
Ammonium sulfate precipitation	49.4	43.1	0.87	106	7.8
DEAE chromatography	14.9	10.0	0.67	25	6.0

### Identification of NecC

**Fig. 3:**
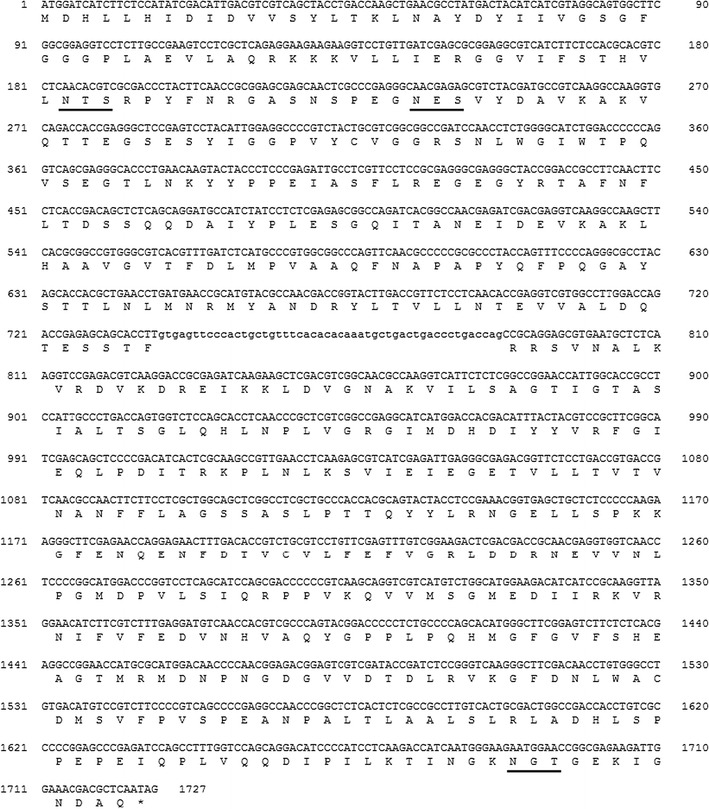
The sequence of NecC from *T. discophora*. Coding DNA sequence and introns are shown in *capital letters* and *lowercase letters*, respectively. The deduced amino acid sequence is indicated *below the coding sequence*. Potential N-linked glycosylation sites of the conserved sequence, NX (S/T), are *underlined*

LC–MS/MS analyses of tryptic digests of NecC for the double bands in SDS–PAGE were conducted to identify its partial amino acid sequences. Nine partial sequences were estimated from the two bands. According to these sequences, five forward and reverse degenerated oligonucleotide primer pairs were designed for PCR amplification. PCR products of estimated sizes were generated with three of the five primer pairs, followed by nested PCR and then they were cloned and sequenced. It was confirmed that several clones had sequences estimated by the LC–MS/MS analyses. Next, a cosmid containing *necC* genomic DNA was retrieved by screening the *T. discophora* genomic DNA library using the *necC* fragment as a probe. Sequence analysis of the cosmid revealed the presence of the whole *necC* genomic DNA sequence (Fig. 3). Comparison of *necC* genomic DNA with *necC* cDNA indicated that one intron, with a length of 50 bp, interrupted the coding region. The intron shared fungal consensus sequences for 5′-splice region, 5′-GTDHSY (D = A, G, or T; H = A, C, or T; S = C or G; Y = C or T), and 3′-splice region, 5′-YAG (Ballance 1986). NecC encodes an open reading frame (ORF) for a protein of 558 amino acids with a calculated molecular mass of 61.3 kDa, which is close to the observed molecular mass based on SDS–PAGE analysis (Fig. 2), and a calculated isoelectric point of 5.5. It contains three potential N-linked glycosylation sites at Asn-62, -79, and -547, according to the conserved sequence (Asn-X-Ser/Thr) (Kukuruzinska et al. 1987).

A homology search of sequence databases using the BLAST program (Altschul et al. 1990) demonstrated that the deduced amino acid sequence of *necC* cDNA had about 67 % of identity to putative fungal glucose-methanol-choline (GMC) oxidoreductases which were hypothetical or genome-derived prediction, and it had a relatively low identity and similarity to proteins that the structures have been experimentally determined. Adenosine diphosphate (ADP)-binding and substrate-binding domains, however, were specifically hit by a search of NecC against NCBI’s conserved domain database (Marchler-Bauer et al. 2005). The ADP-binding βαβ-fold motif of GXGXXG near N-terminus, which is conserved among FAD-binding proteins such as GMC oxidoreductases (Wierenga et al. 1986; Kiess et al. 1998; Dijkman et al. 2013), was found in the NecC amino acid residues 27–32, namely GSGFGG.

### Bacterial expression of NecC

The coding region of *necC* was inserted into pET vector and expressed by *E. coli* BL21 as C-terminal His-tagged protein, and was then purified by Ni Sepharose column chromatography. This protein gave a single band for a molecular mass of about 62 kDa as compared with marker proteins in SDS–PAGE (data not shown) which corresponded to the calculated molecular mass of the His-tagged NecC. Incubation of 4-amino-4-deoxyarabinitol at 15 °C for 12.5 h with the recombinant NecC led to an increase in the amount of nectrisine whose identity was confirmed by its molecular mass (in the Additional file 1: Fig. S1), however, the incubation without the enzyme did not cause an increase in the amount of nectrisine, and the 4-amino-4-deoxyarabinitol remained unchanged. This indicated that the recombinant NecC had 4-amino-4-deoxyarabinitol oxidase activity, the same as the NecC from *T. discophora*, thus providing verification of the *necC* gene.

### Properties of NecC

**Fig. 4:**
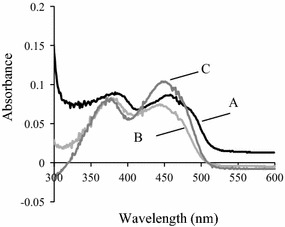
Adsorption spectra of NecC from *T. discophora* before and after precipitation with TCA. Spectra of the untreated enzyme in PBS, pH 7.4 (*A*), supernatant after the precipitation of the enzyme (*B*) and FAD standard in PBS containing 4.8 % TCA (*C*) are shown

The purified NecC from *T. discophora* showed yellow color based on visual inspection, suggesting that the enzyme contained a cofactor. The absorption spectrum of the native enzyme showed the characteristics of flavoprotein absorption spectrum (Ghisla 1980) with maxima at 272, 388 and 457 nm, as well as a shoulder at around 480 nm (Fig. 4). To determine whether the cofactor was covalently bound to NecC or not, precipitation of NecC with TCA was performed and spectra before and after the precipitation were analyzed. The spectrum of the supernatant from 300 to 550 nm showed similar pattern to that of the untreated enzyme, with the absorbance being almost at the same level as that of the untreated enzyme, indicating that most of the cofactor was released from the enzyme by the TCA treatment. This suggested that the cofactor was non-covalently bound to the enzyme. It was also found that the cofactor had a spectrum similar to that of FAD in the same buffer, which suggested that the cofactor could contain a flavin moiety.

**Fig. 5:**
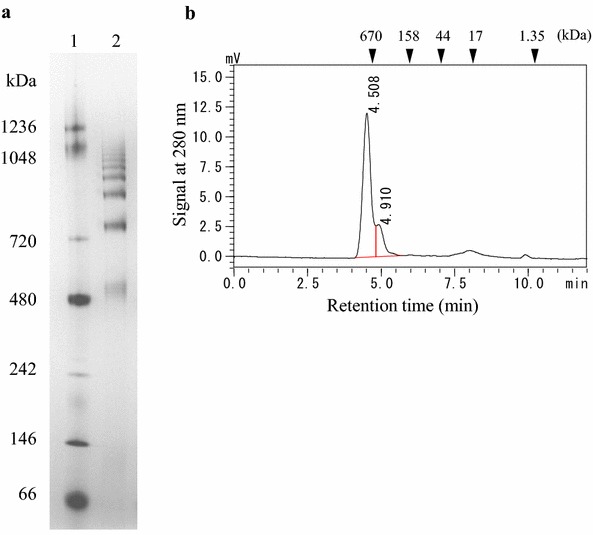
Blue native PAGE (**a**) and size-exclusion chromatogram (**b**) of NecC protein produced by *T. discophora*. *Lane 1* marker proteins; *lane 2* NecC. *Triangles* in **b** indicate elution positions of marker proteins

As described above, NecC from the fungus showed bands at around 60 kDa in SDS–PAGE (Fig. 2), however, it showed at least six bands in native PAGE (Fig. 5) ranging from about 500 to 1000 kDa. This suggested that NecC formed several non-covalently linked oligomers. The 500, 750, and 880 kDa bands shown in the native PAGE were likely to correspond to an octamer, dodecamer, and tetradecamer, respectively, since the NecC monomer had a calculated molecular mass of 61.3 kDa. The oligomeric state of NecC was also analyzed by SE-HPLC using an Acquity UPLC BEH200 column. A major peak eluted at the almost void volume, with the molecular mass being estimated to be above 670 kDa, which may correspond to the bands above 750 kDa in the native PAGE gel. A second peak at around 500 kDa in the chromatogram is thought to correspond to the band of 500 kDa in the native PAGE gel.

### Enzyme assay of NecC

**Fig. 6:**
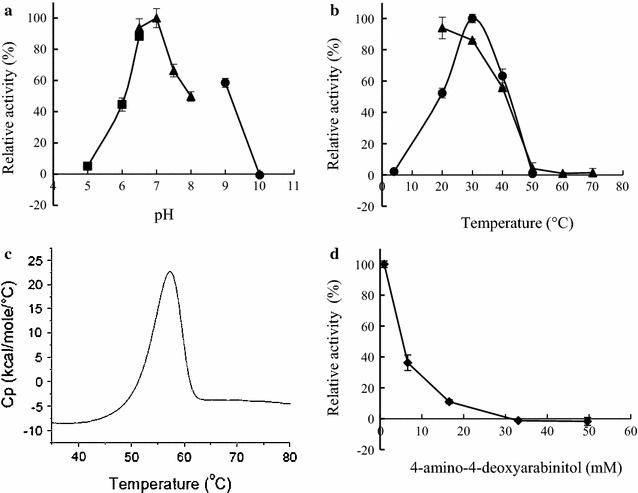
Effect of pH, temperature and 4-amino-4-deoxyarabinitol concentration on NecC activity. **a** The enzyme activity was measured under standard assay conditions except for pH. *closed square*, citrate/phosphate buffer; *closed triangle*, phosphate buffer; *closed circle*, glycine/NaOH buffer. **b** The enzyme activity was measured under standard assay conditions at various temperatures (*closed circle*). For the stability study, the enzyme in 50 mM potassium phosphate buffer pH 7.0 was preincubated for an hour at the indicated temperature, then the remaining activity was measured under the standard condition (*closed triangle*). **c** DSC thermogram of NecC in PBS, pH 7.4. **d** The enzyme activity was measured under standard assay conditions except for 4-amino-4-deoxyarabinitol concentration

The optimal pH for the NecC activity was indicated to be pH 7.0 (Fig. 6a). NecC activity could not be detected under pH 5 and above pH 10 under standard assay conditions. Figure 6b shows the effect of temperatures ranging from 4 to 70 °C on the NecC activity. Maximum activity was obtained at 30 °C. Activity could not be detected at 4 °C and above 60 °C, and was only slightly detected at 50 °C under standard assay conditions. Thermal stability for the activity was determined by measuring the activity after incubation of the enzyme solution for 1 h at temperatures ranging from 20 to 70 °C. The activity was almost stable up to 30 °C, slightly detected at 50 °C, and was totally inactivated above 60 °C. To confirm the conformational stability of NecC against heat, thermal unfolding of NecC in PBS was measured by DSC (Fig. 6c). Onset of the DSC curve was around 45 to 50 °C and the melting temperature, Tm, of NecC was 57.3 °C under the assay conditions. These results indicated that the inactivation of NecC activity by heat was presumably due to unfolding of the enzyme.

Next, the effect of the substrate concentration on NecC activity was investigated. An increase in the concentration of 4-amino-4-deoxyarabinitol led an inhibition of NecC activity (Fig. 6d). The activity could not be detected at a 4-amino-4-deoxyarabinitol concentration of 33 mM. NecC also exhibited activity against some sugar alcohols in the absence of 4-amino-4-deoxyarabinitol. The ratios of the relative activities of d-sorbitol, d-arabinitol and xylitol to 4-amino-4-deoxyarabinitol were 46, 47, and 48 %, respectively, indicating that 4-amino-4-deoxyarabinitol was the preferred substrate. NecC activity with 1 mM of 4-amino-4-deoxyarabinitol was not inhibited by the coexistence of 1 mM of the sugar alcohols (Table 2). The activity was also virtually unchanged by 1 mM of Na^+^, Mg^2+^, K^+^, Ca^2+^, Co^2+^, Ni^2+^ or Zn^2+^, inhibited by 1 mM of Cu^2+^ or chelating reagent, ethylenediaminetetraacetic acid (EDTA), and slightly enhanced by Mn^2+^ (Table 2). Addition of 0.2, 1 or 5 mM Mn^2+^ enhanced the enzyme activity by 20, 27 or 30 %, respectively.

**Table 2:** Effect of additives on NecC activity

Additives	Relative activity (%)
None	100
D-sorbitol	98
D-arabinitol	97
Xylitol	101
NaCl	97
MgCl_2_	102
KCl	101
CaCl_2_	100
MnCl_2_ (0.2 mM)	120
MnCl_2_ (1 mM)	127
MnCl_2_ (5 mM)	130
CoCl_2_	92
NiCl_2_	89
CuCl_2_	73
ZnCl_2_	99
EDTA	40

The enzyme activities were measured under standard assay conditions containing 1 mM 4-amino-4-deoxyarabinitol with 1 mM per additives, unless otherwise specifically noted. Values are an average of three independent experiments

### Accumulation of 4-amino-4-deoxyarabinitol in *T. discophora*

**Fig. 7:**
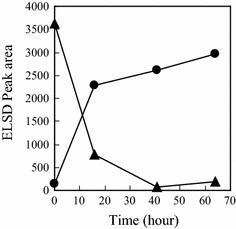
Incubation of filtrated *T. discophora* extracts. Relative amounts of nectrisine (*closed circle*) and 4-amino-4-deoxyarabinitol (*closed triangle*) were shown

When extraction from *T. discophora* mycelium was performed at a small scale (~1 mL), a small amount of 4-amino-4-deoxyarabinitol compared with nectrisine was detected in the extracts. It was observed, however, that extraction at a 6 kL scale led to a great increase in the amounts of 4-amino-4-deoxyarabinitol. To clarify the accumulation of 4-amino-4-deoxyarabinitol in the extracts, the NecC activity in the extracts was immediately suppressed just after extraction by cooling in an ice-water bath. In the extracts (Fig. 7 at 0 min), it was notable that the amount of 4-amino-4-deoxyarabinitol was abundant and nectrisine was found in trace amounts, which suggested that not nectrisine but 4-amino-4-deoxyarabinitol may be mainly accumulated in the cells. Incubation of the extracts led to a decrease in the amount of 4-amino-4-deoxyarabinitol along with an increase in the amount of nectrisine, which was presumably catalyzed by NecC (Fig. 7). This indicated that NecC could be extracted by the heat treatment in an aqueous slurry of the culture broth.

## Discussion

In our previous study, a biosynthetic pathway to an antibiotic nectrisine in *T. discophora* was proposed via d-xylulose 5-phosphate and 4-amino-4-deoxyarabinitol, and it was found that the crude enzyme converted 4-amino-4-deoxyarabinitol to nectrisine (Miyauchi et al. 2015). In the current study, we aimed to characterize the enzyme responsible for the conversion of 4-amino-4-deoxyarabinitol to nectrisine. A novel oxidase, NecC, was first purified and showed characteristics of an FAD-dependent GMC oxidoreductase family. Ammonium sulfate fractionation was effective for the enzyme purification and excellent recovery of the enzyme activity. Subsequent anion exchange chromatography increased the protein purity of NecC based on SDS–PAGE analysis, however, slightly decreased its specific activity. This may have been caused by the partial release of its cofactor at the chromatography step. Washing the column to which the enzyme bound could have resulted in the partial release of the cofactor from the enzyme since the cofactor was indicated to be non-covalently bound to the enzyme (Fig. 4). In general, GMC oxidoreductases form oligomers, such as glucose oxidases of homodimer (Frederick et al. 1990; Kiess et al. 1998), alcohol oxidases of eight identical subunits (Kato et al. 1976; Vonck and van Bruggen 1990; Boteva et al. 1999; Soldevila et al. 2000; Ozimek et al. 2005), and pyranose oxidases of four identical subunits (Giffhorn 2000; Wongnate and Chaiyen 2013). These reports suggested that these oxidases under native conditions were virtually uniform in size. However, several size variants were detected of NecC produced by *T. discophora* under native conditions. On the other hand, under denaturing conditions, NecC showed double bands at around 60 kDa in SDS–PAGE. This was probably due to partial glycosylation which is known to migrate bands and affect molecular weight determinations in SDS–PAGE (Segrest and Jackson 1972), since potential N-glycosylation sites were found in the sequence. Moreover, bacterially expressed NecC had the activity and exhibited a single band in SDS–PAGE (data not shown) which indicated that NecC protein could be derived from the homogeneous sequence. Therefore, the oligomer of NecC could be composed of identical subunits in terms of the amino acid sequence.

**Fig. 8:**
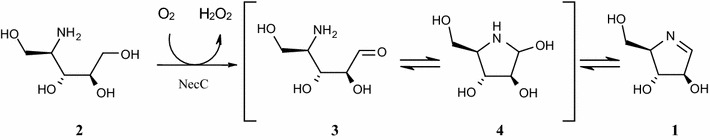
Postulated scheme for reaction catalyzed by NecC

NecC converts 4-amino-4-deoxyarabinitol to nectrisine in aqueous solution without additional compounds such as nicotinamide adenine dinucleotide (NAD). Thus, O_2_ is expected to be an electron accepter and H_2_O_2_ is generated in the reaction. This is followed by the facts that typical GMC oxidases reduce O_2_ to H_2_O_2_ (Wongnate and Chaiyen 2013; Dijkman et al. 2013) and, in the enzyme activity assay, ABTS was oxidized with the NecC reaction mixture and peroxidase, which indicated that the NecC reaction mixture was likely to contain H_2_O_2_. From these findings, the NecC reaction could be postulated as shown in Fig. 8. NecC should catalyze oxidation of 4-amino-4-deoxyarabinitol to the intermediate **3** concomitant with reduction of O_2_ to H_2_O_2_. Subsequently, spontaneous intramolecular addition of the amino group to the carbonyl group of **3** is reasonably expected to occur, resulting in the cyclic compound **4**, with dehydration to afford nectrisine. Potential intermediates, **3** and **4**, however, could not be detected by LC–MS analysis in the NecC reaction mixture, indicating that nectrisine was the main molecule generated.

The antibiotic nectrisine might play a role in protecting the fungus from bacterial attack like a β-pyrone antibiotic cortalcerone. Cortalcerone can be produced by several white rot fungi, and pyranose oxidase, which belongs to the GMC oxidoreductase family, is involved in its biosynthesis (Artolozaga et al. 1997; Giffhorn 2000; Pazarlioglu et al. 2012; Wongnate and Chaiyen 2013), which could be considered to be analogous to the case of nectrisine production by *T. discophora*.

It is important to understand the step of the nectrisine extraction from the fungus in order to develop an industrial manufacturing process for nectrisine. The extraction step of nectrisine was thus studied as shown above, and a description about the possible generation of nectrisine could be made as follows: first, 4-amino-4-deoxyarabinitol and NecC are extracted with heat treatment from the culture broth, then, in vitro, 4-amino-4-deoxyarabinitol is converted to nectrisine catalyzed by NecC with oxygen consumption and H_2_O_2_ generation. This similar heat treatment was reported for extraction of a pyranose oxidase from the mycelium of a white rot fungus (Schafer et al. 1996). We believe that these findings are helpful in establishing a nectrisine manufacturing process with *T. discophora*, especially at a large scale.

## Additional file

10.1186/s13568-016-0176-1 Conversion of 4-amino-4-deoxyarabinitol to nectrisine with His-tagged NecC expressed by recombinant *E. coli*. **a**, **b**, and **c**, Absorbance chromatograms; **d**, **e**, and **f**, the extracted MS chromatograms for *m/z* 297.1 which is the [M+H]^+^ ion for reduced and NBD-labeled nectrisine. Conversions of 4-amino-4-arabinitol in the presence (**a** and **d**) or absence (**b** and **e**) of the recombinant NeccC are shown. For reference, chromatograms of reduced and NBD-labeled nectrisine which is equal to NBD-labeled 1,4-Dideoxy-1,4-imino-D-arabinitol are depicted in **c** and **f**.
