# Supplementary material for: Characterization of a novel oxidase from Thelonectria discophora SANK 18292 involved in nectrisine biosynthesis
Source: AMB Express. 2016 Jan 20;6:6. doi: 10.1186/s13568-016-0176-1 (PMC4718913; doi:10.1186/s13568-016-0176-1)
Supplement: Supplementary file 1 — 10.1186/s13568-016-0176-1 Conversion of 4-amino-4-deoxyarabinitol to nectrisine with His-tagged NecC expressed by recombinant E. coli. a, b, and c, Absorbance chromatograms; d, e, and f, the extracted MS chromatograms for m/z 297.1 which is the [M+H]+ ion for reduced and NBD-labeled nectrisine. Conversions of 4-amino-4-arabinitol in the presence (a and d) or absence (b and e) of the recombinant NeccC are shown. For reference, chromatograms of reduced and NBD-labeled nectrisine which is equal to NBD-labeled 1,4-Dideoxy-1,4-imino-D-arabinitol are depicted in c and f. [file 13568_2016_176_MOESM1_ESM.docx]

AMB Express

Characterization of a novel oxidase from *Thelonectria discophora* SANK 18292 involved in nectrisine biosynthesis

Ryuki Miyauchi^1,^*, Hidetaka Sakurai^2^, and Yoichiro Shiba^3^

^1^ New Modality Research Laboratories, R&D Division, Daiichi Sankyo Co., Ltd., 1-2-58, Hiromachi, Shinagawa-ku, Tokyo 140-8710, Japan.

^2^ Discovery Science and Technology Department, Drug Discovery and Biomedical Technology Unit, Daiichi Sankyo RD Novare Co., Ltd., 1-16-13, Kitakasai, Edogawa-ku, Tokyo 134-8630, Japan.

^3^ CM&C Planning Department, Pharmaceutical Technology Division, Daiichi Sankyo Co., Ltd., 1-12-1, Shinomiya, Hiratsuka-shi, Kanagawa 254-0014, Japan.

* Corresponding author

Ryuki Miyauchi

New Modality Research Laboratories, R&D Division, Daiichi Sankyo Co., Ltd., 1-2-58, Hiromachi, Shinagawa-ku, Tokyo 140-8710, Japan.

e-mail: [miyauchi.ryuki.d4@daiichisankyo.co.jp](mailto:miyauchi.ryuki.d4@daiichisankyo.co.jp)

Tel: +81 3 3492 3131

Fax: +81 3 5740 3643

Reduced,

NBD-labeled **1**

NBD-labeled **2**

Reduced,

NBD-labeled **1**

**e**

**f**

**d**

**c**

**b**

**a**

Absorbance

*m/z* 297.1

Intensity (10^6^)


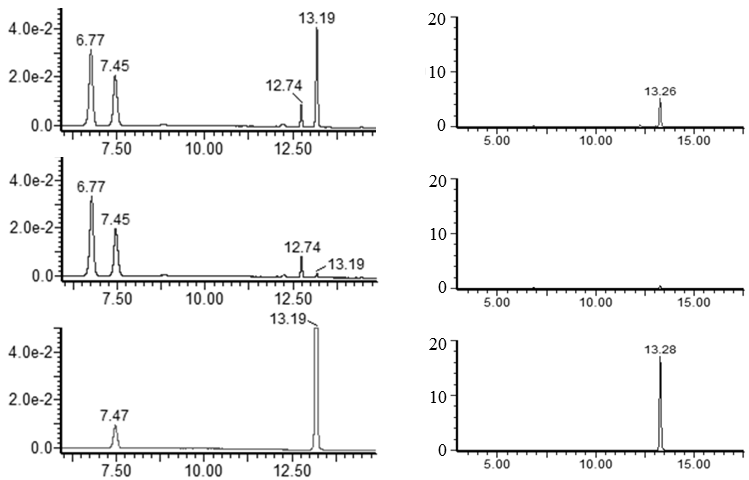


Signal at 500 nm

Time (min)

Time (min)

Fig. S1 Conversion of 4-amino-4-deoxyarabinitol to nectrisine with His-tagged NecC expressed by recombinant *E. coli*. a, b, and c, Absorbance chromatograms; d, e, and f, the extracted MS chromatograms for *m/z* 297.1 which is the [M+H]^+^ ion for reduced and NBD-labeled nectrisine. Conversions of 4-amino-4-arabinitol in the presence (a and d) or absence (b and e) of the recombinant NecC are shown. For reference, chromatograms of reduced and NBD-labeled nectrisine which is equal to NBD-labeled 1,4-Dideoxy-1,4-imino-D-arabinitol are depicted in c and f
